# *Mycobacterium tuberculosis* transmission in an ethnically-diverse high incidence region in England, 2007–11

**DOI:** 10.1186/s12879-018-3585-8

**Published:** 2019-01-07

**Authors:** Emilia Vynnycky, Adrienne R. Keen, Jason T. Evans, Shaina Khanom, Peter M. Hawkey, Richard G. White, Ibrahim Abubakar

**Affiliations:** 1 Statistics, Modelling and Economics Department, 61 Colindale Avenue, Colindale, London, NW9 5HT UK; 2 TB Modelling Group, Centre for Mathematical Modelling of Infectious Diseases, TB Centre and Faculty of Epidemiology and Population Health, London School of Hygiene and Tropical Medicine, London, UK; 3 PHE West Midlands Public Health Laboratory, Heart of England NHS Foundation Trust, Birmingham, UK; 4 Public Health Wales Microbiology Cardiff, Llandough Hospital, Penlan Road, Penarth, CF64 2XX UK; 5 Institute of Microbiology and Infection, College of Medical and Dental Sciences, University of Birmingham, Edgbaston, Birmingham, B15 2TT UK; 6 Queen Elizabeth Hospital, Birmingham, B15 2TH UK; 7 Research Department of Infection and Population Health, University College London, London, UK

**Keywords:** Tuberculosis, West midlands, England, MIRU-VNTR, Clustering, Transmission, Contact patterns

## Abstract

**Background:**

Transmission patterns in high tuberculosis incidence areas in England are poorly understood but need elucidating to focus contact tracing. We study transmission within and between age, ethnic and immigrant groups using molecular data from the high incidence West Midlands region.

**Methods:**

Isolates from culture-confirmed tuberculosis cases during 2007–2011 were typed using 24-locus Mycobacterial Interspersed Repetitive Unit-Variable Number Tandem Repeats (MIRU-VNTR). We estimated the proportion of disease attributable to recent transmission, calculated the proportion of isolates matching those from the two preceding years (“retrospectively clustered”), and identified risk factors for retrospective clustering using multivariate analyses. We calculated the ratio (RCR) between the observed and expected proportion clustered retrospectively within or between age, ethnic and immigrant groups.

**Results:**

Of the 2159 available genotypes (79% of culture-confirmed cases), 34% were attributed to recent transmission. The percentage retrospectively clustered decreased from 50 to 24% for 0–14 and ≥ 65 year olds respectively (*p* = 0.01) and was significantly lower for immigrants than the UK-born. Higher than expected clustering occurred within 15–24 year olds (RCR: 1.4 (95% CI: 1.1–1.8)), several ethnic groups, and between UK-born or long-term immigrants with the UK-born (RCR: 1.8 (95% CI: 1.1–2.4) and 1.6 (95% CI: 1.2–1.9) respectively).

**Conclusions:**

This study is the first to consider “who clusters with whom” in a high incidence area in England, laying the foundation for future whole-genome sequencing work. The higher than expected clustering seen here suggests that preferential mixing between some age, ethnic and immigrant groups occurs; prioritising contact tracing to groups with which cases are most likely to cluster retrospectively could improve TB control.

**Electronic supplementary material:**

The online version of this article (10.1186/s12879-018-3585-8) contains supplementary material, which is available to authorized users.

## Background

Recently, tuberculosis notification rates in England (10.5/100,000 in 2015) have been the highest in Western Europe, with the highest notification rates occurring in London and urban centres, including the West Midlands [1]. Over 70% of cases in England occur in the foreign-born [1], but little is known about the amount of ongoing transmission between different subgroups in the population, particularly in high incidence areas. Understanding transmission patterns and “who infects whom” is important for improving control, as it can help direct contact tracing to the most likely sources of infection.

Molecular strain-typing data on “who is clustered with whom” may provide insight into transmission patterns. Cases whose isolates share identical strain types are said to be “clustered”. In England, TB strain-typing has been conducted using 24-locus Mycobacterial Interspersed Repetitive Unit-Variable Number Tandem Repeats (MIRU-VNTR) since 2010. Clustering may occur if cases belong to the same transmission chain but could also result from a common strain-type circulating in England or elsewhere. Molecular studies in England to date, based on MIRU-VNTR, have typically considered short time periods (2010–2012) [2, 3], the amount of household transmission and risk factors for clustering, but have not yet studied the characteristics of cases with whom different cases cluster. The latter depends largely on transmission patterns, and is affected by other factors, including disease susceptibility. A study in Oxfordshire [4] considered a 5 year period (2007–2012) using whole-genome sequencing (WGS), which has a higher resolution than MIRU-VNTR [5]. However, this study considered a low tuberculosis incidence area (notification rate of 8/100000/year in 2016) and WGS has not yet been used in England to study *M tuberculosis* transmission in high incidence areas.

Using 24-locusMIRU-VNTRstrain-typing data for 2007–2011 from the West Midlands, a high incidence ethnically diverse area in England (notification rates of 18 and 12/100,000/year in 2011 and 2015 respectively), we combine risk factor analyses with analyses of “who is clustered with whom” to get insight into transmission patterns by age, ethnic and immigrant group and discuss some implications for contact tracing.

## Methods

### Study population

The study population included all culture-confirmed tuberculosis cases from the West Midlands region, notified during 1st January 2007-31st December 2011, with an eligible 15 or 24-locusMIRU-VNTRstrain-type (see below). The region numbers 5.6 million residents [6], including several cities with > 500,000 residents (e.g. Birmingham, Coventry, and Wolverhampton).

### Molecular data and clustering definitions

During 2007–2009, culture-positive isolates were routinely typed with a set of 15 MIRU-VNTR loci [7]. From 2010, nine additional loci were typed [8] using the internationally-recommended set of 24 MIRU-VNTR loci. To extend the dataset of 24-locus profiles, whilst conserving laboratory resources, strains isolated during 2007–2009 which clustered in a preliminary analysis (isolates matching identically on at least 14 out of 15 loci) using all isolates from 2007 to 11 were typed with the additional nine loci. Isolates were then included in the present study if: 1) they had a unique 14 or 15 locus MIRU-VNTR profile (unclustered on the preliminary analysis) or 2) their 24-locus MIRU-VNTR profile had at least 23 loci typed. Cases notified during 1st January 2009-31st December 2011 whose isolate matched identically on 24-locus typing with that from a case notified up to 2 years previously were defined to be “clustered respectively”.

### Data collection

Data on notified cases are held in the national Enhanced TB Surveillance (ETS) database, which contains patient-level demographic data (age, sex, world region of birth, ethnic group, and time from entry to the UK and tuberculosis diagnosis for foreign-born individuals), clinical details (including disease site and notification year), behavioural risk factors (history of/current problem drug or alcohol use and history of/current homelessness or time spent in prison); laboratory data (culture-positivity and drug sensitivity). Clinical specimens and referred cultures from suspected tuberculosis cases in the West Midlands were routinely sent to the Regional Centres for Mycobacteriology, Birmingham, for culturing, identification, strain-typing, and drug susceptibility testing using standard methods [9]. Strain types and other laboratory data were matched to patient-level ETS data [10]. Duplicate notifications and specimens from the same patient occurring within 12 months of initial notification or specimen-collection were collated. TB episodes more than 12 months apart were considered separate notifications.

### Data and risk factor analysis

We estimated the proportion of cases during 2007–2011 attributable to recent transmission using the “n-1” method [11], implicitly assuming that one source case initiates each cluster, and compared the estimate against the proportion of cases notified during 2009–2011 that were clustered retrospectively. In sensitivity analyses we compared estimates of the proportion attributed to recent transmission for the “n-1” method using different time windows (2007–9, 2007–2010 and 2007–2011) and compared that against the proportion retrospectively clustered with other cases during the preceding 2 years for the same time window.

The proportion retrospectively clustered was also calculated for the demographic characteristics, clinical details, behaviour risk factors described above and drug sensitivity. We conducted a univariate analysis of factors associated with retrospective clustering and report maximum likelihood estimates of odds ratios (OR) with Wald tests with 95% confidence limits. Significance was evaluated using *p*-values from the likelihood ratio chi-square test (LRT), with *p* < 0.05 considered significant.

Multivariate logistic regression models were also constructed, including the age group, sex and other variables significantly associated with clustering in the univariate analysis. Either the region of birth or ethnicity were included, with region of birth preferred if both were significant. To avoid reducing models to just those foreign-born, time since entry in the UK was excluded in multivariate models, as were behavioural risk factors, which were collected for only some cases. For factors included in multivariate models, adjusted ORs and their 95% confidence limits were reported, with significance evaluated using *p*-values from the LRT. For consistency with other risk factor studies of clustering [2, 3] cases clustered retrospectively just with extrapulmonary cases were included. However, they were excluded in subsequent analyses.

### Analyses of who’s clustered with whom

To get insight into possible age-specific sources of infection, we calculated the proportion of cases notified during 2009–2011 in each age group (0–4, 5–14, 15–24, 25–34, 35–44, 45–54, 55–64, 65–74 and ≥ 75 years), that were clustered retrospectively with pulmonary cases in given age groups. For cases aged 15–24 years, for example, the proportion retrospectively clustered with pulmonary cases aged *j* was given by:

$$ \frac{C_{15-24,j}}{\sum_{i=1}^9{C}_{15-24,i}}\times \frac{R_{15-24}}{N_{15-24}} $$

where *C*_15 − 24, *j*_ is the number of pulmonary cases aged *j* with whom cases aged 15–24 years notified during 2009–11 were clustered retrospectively, *R*_15 − 24_ is the number of cases aged 15–24 years during 2009–11 who were clustered retrospectively with pulmonary cases of any group, and *N*_15 − 24_ is the total number of cases aged 15–24 years notified during 2009–11.

Adapting published methods [12], we calculated the retrospective clustering ratio (RCR), defined as the ratio between the proportion of retrospectively clustered cases in each age group that were clustered with pulmonary cases aged *j*, and that expected, according to proportionate mixing. For this assumption, the probability of retrospective clustering with given age groups depends only on how many pulmonary cases in those age groups were notified up to 2 years before the given case. Considering cases aged 15–24 years, for example, the ratio is given by:

$$ \frac{C_{15-24,j}}{\sum_{i=1}^9{C}_{15-24,i}}/\frac{T_{15-24,j}}{\sum_{i=1}^9{T}_{15-24,i}} $$

where *T*_15 − 24, *j*_ is the total number of pulmonary cases aged *j* notified during the 2 years before the *N*_15 − 24_ cases aged 15–24 years who were notified during 2009–11. Values for the ratio exceeding and below 1 suggest that there is more and less clustering respectively than expected between cases in given age groups. Confidence intervals were constructed through bootstrapping, using 10,000 bootstrap-derived datasets, generated by sampling clusters with replacement based on Borgdorff et al. [13]. Clusters appearing multiple times in a bootstrap dataset were treated as independent.

The proportion retrospectively clustered and the RCR were analysed similarly considering different ethnic groups, the UK-born and immigrants by time since arrival in the UK. In sensitivity analyses, the proportion retrospectively clustered and the retrospective clustering ratio were calculated using time windows of 3 and 4 years to assess retrospective clustering. In these calculations, cases who were notified during the periods 2007–9 and 2007–10 respectively were not eligible to be retrospectively clustered.

### Software

Risk factor analyses were conducted using Stata/SE 13.1 (StataCorp LP); other analyses were conducted using a specially-written C program with published routines [14].

## Results

### Study population and descriptive analysis

**Fig. 1:**
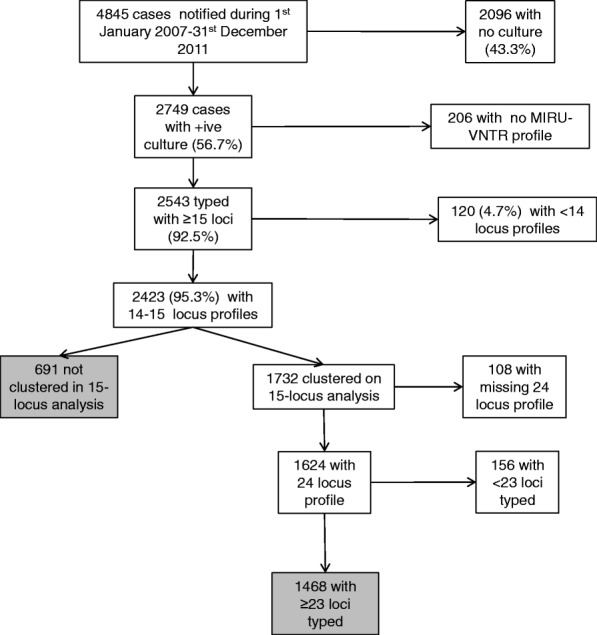
Description of tuberculosis cases in the West Midlands during 2007–11 and their genotyping results. The grey boxes refer to cases who were included in the analyses based on 24-locus MIRU-VNTR. The sum of the cases in these grey boxes therefore gives the 2159 cases that were eligible for risk factor analysis mentioned in the text

During 1st January 2007-31st December 2011, 4845 clinical tuberculosis cases were notified in the West Midlands region. 2749/4845 (56.7%) were culture-positive, and 2543/2749 (92.5%) isolates were typed with at least 15 loci (Fig. 1). The cases with and without isolates typed had similar demographic characteristics (Table 1). Of those typed, 2423/2543 (95.3%) were eligible for preliminary cluster analyses (Fig. 1). These identified 691 cases who were not clustered and 1732 clustered cases, of which 1468 had at least 23 loci typed, resulting in 2159 (=691 + 1732) cases eligible for risk factor analysis for clustering using 24-locus profiles.

**Table 1:** Characteristics of all 4845 cases notified in the West Midlands (2007–2011) and the study population

	All cases		Cases with genotype data		Cases without genotype data	
	Number	%	Number	%	Number	%
Year notified						
2007	938	19.4	524	20.6	414	18
2008	1015	21	502	19.7	513	22.3
2009	1009	20.8	536	21.1	473	20.5
2010	872	18	494	19.4	378	16.4
2011	1011	20.9	487	19.2	524	22.8
Sex						
Male	2638	54.5	1414	56	1224	53
Female	2205	45.5	1127	44	1078	47
Age group (years)						
0–14	286	5.9	62	2.4	224	9.7
15–44	2769	57.2	1617	63.6	1152	50
45–64	966	19.9	470	18.5	496	21.5
65 and over	824	17	394	15.5	430	18.7
Region of birth						
UK	1555	34.8	754	31.7	801	38.3
Europe	101	2.3	57	2.4	44	2.1
East Mediterranean	45	1	27	1.1	18	0.9
Africa	700	15.7	417	17.5	283	13.5
Americas	57	1.3	30	1.3	27	1.3
South Asia	1912	42.8	1033	43.4	879	42
East/Southeast Asia	103	2.3	64	2.7	39	1.9
Ethnicity						
White	880	18.8	412	16.8	468	21.1
Black-Caribbean	173	3.7	96	3.9	77	3.5
Black-African	711	15.2	406	16.5	305	13.7
Black-Other	18	0.4	12	0.5	6	0.3
South Asian	2602	55.6	1364	55.5	1238	55.7
Chinese	47	1	21	0.9	26	1.2
Mixed/Other	248	5.3	145	5.9	103	4.6
Years since entry to tuberculosis diagnosis						
0–1	435	16.4	262	17.6	173	14.8
2–4	573	21.5	357	24	216	18.4
5–9	606	22.8	349	23.4	257	21.9
10 and over	1046	39.3	521	35	525	44.8
Disease site						
Pulmonary, with or without extra-pulmonary	2632	55.1	1635	64.6	997	44.4
Extra-pulmonary only	2141	44.9	895	35.4	1246	55.6
History of or current problem drug use						
No	2181	96.8	1126	96.1	1055	97.6
Yes	72	3.2	46	3.9	26	2.4
History of or current problem alcohol use						
No	2138	97.3	1100	96.6	1038	98.1
Yes	59	2.7	39	3.4	20	1.9
History of or current homelessness						
No	2201	98.1	1144	97.0	1057	99.2
Yes	43	1.9	35	3.0	8	0.8
History of or currently in prison						
No	2084	97.1	1079	96	1005	98.3
Yes	62	2.9	45	4.0	17	1.7

### Risk factors for retrospective clustering

Of the 2159 cases analysed, 959 isolates (44%, 95% CI: 42–46%) shared identical genotypes during 2007–11, comprising 225 clusters, with 119 including two cases and 77, 16 and 9 clusters with 3–5, 6–9 and 11–49 cases respectively. Only one cluster, with 102 cases, had > 50 cases.

**Fig. 2:**
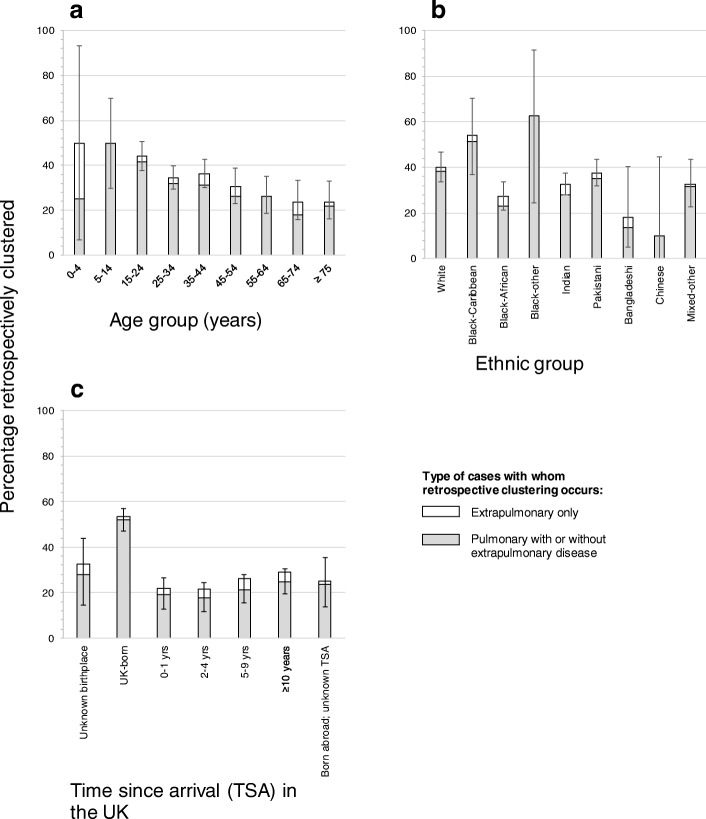
Percentage of cases by **a**. age, **b**. ethnic and **c**. immigrant group notified during 2009–11 that are retrospectively clustered. The shaded and unshaded areas show the proportion retrospectively clustered with pulmonary cases, with or without extrapulmonary disease and extrapulmonary cases respectively

Of cases notified during 2009–2011, 452/1329 (3%, 95% CI: 31–37%) were clustered retrospectively, which was similar to the percentage of cases during 2007–2011 attributed to recent transmission using the “n-1” method ((959–225)/2159 = 734/2159 or 34% (95% CI: 32–36%)). Most of the retrospective clustering occurred with pulmonary cases (Fig. 2). The percentage retrospectively clustered was relatively insensitive to the study period, whilst the percentage of cases attributed to recent transmission decreased as the duration of the study period decreased, to 32% (95% CI: 30–34) and 30% (95% CI: 28–33) considering the period 2007–10 and 2007–9 respectively (Table 3).

The percentage clustered retrospectively was similar for males and females (Table 2), decreasing with increasing age from 50% for 0–14 year olds to 24% for those aged at least 65 years (OR: 1.00 vs 0.3 (95% CI: 0.1–0.7), *p* < 0.001). A high percentage (53%) of UK-born cases were clustered retrospectively, compared to those born abroad (e.g. 26% of those born in South East Asia, OR: 0.3, 95%CI: 0.3–0.4, *p* < 0.001). The percentage clustered retrospectively varied between ethnic groups (27, 34, 40 and 54% for the Black African, South Asian, White and Black-Caribbean populations respectively (*p* = 0.01)).

**Table 2:** Demographic features and risk factors for clustering using 24-locus typing for cases notified in the West Midlands, by the retrospective method of clustering

	All cases, 09–11		Clustered cases					
	N	Col %	N	%	OR (95% CI)	*P*	aOR (95% CI)	*p*
Sex								
Male	744	56	267	35.9	1		1	
Female	585	44	185	31.6	0.8 (0.7,1)	0.1	0.8 (0.6,1)	0.03
Total	1329	100	452	34				
Age group (years)								
0–14	30	2.3	15	50	1		1	
15–44	830	62.5	313	37.7	0.6 (0.3,1.3)	0.18	0.8 (0.4,1.7)	0.51
45–64	263	19.8	75	28.5	0.4 (0.2,0.9)	0.02	0.4 (0.2,0.9)	0.04
65 and over	206	15.5	49	23.8	0.3 (0.1,0.7)	< 0.01	0.3 (0.1,0.7)	0.01
Total	1329	100	452	34				
Birthplace								
UK	401	31.5	214	53.4	1		1	
Europe	37	2.9	10	27	0.3 (0.2,0.7)	< 0.01	0.3 (0.1,0.7)	< 0.01
East Mediterranean	16	1.3	4	25	0.3 (0.1,0.9)	0.04	0.2 (0.1,0.8)	0.02
Africa	221	17.3	54	24.4	0.3 (0.2,0.4)	< 0.01	0.3 (0.2,0.4)	< 0.01
Americas	14	1.1	5	35.7	0.5 (0.2,1.5)	0.2	0.7 (0.2,2.3)	0.59
South Asia	554	43.5	145	26.2	0.3 (0.2,0.4)	< 0.01	0.4 (0.3,0.5)	< 0.01
East/Southeast Asia	32	2.5	5	15.6	0.2 (0.1,0.4)	< 0.01	0.2 (0.1,0.4)	< 0.01
Total	1275	100	437	34.3				
Ethnicity								
White	220	17.2	88	40	1			
Black-Caribbean	37	2.9	20	54.1	1.8 (0.9,3.6)	0.11	–	–
Black-African	213	16.7	58	27.2	0.6 (0.4,0.8)	< 0.01	–	–
Black-Other	8	0.6	5	62.5	2.5 (0.6,10.7)	0.22	–	–
South Asian	703	55.1	241	34.3	0.8 (0.6,1.1)	0.12	–	–
Chinese	10	0.8	1	10	0.2 (0,1.3)	0.09	–	–
Mixed/Other	86	6.7	28	32.6	0.7 (0.4,1.2)	0.23	–	–
Total	1277	100	441	34.5				
Time since entry to UK to tuberculosis diagnosis (years)*								
0–1	147	18	32	21.8	1			
2–4	176	21.5	39	22.2	1 (0.6,1.7)	0.93	–	–
5–9	206	25.2	53	25.7	1.2 (0.8,2.1)	0.39	–	–
10 and over	288	35.3	83	28.8	1.5 (0.9,2.3)	0.12	–	–
Total	817	100	207	25.3				
Disease site								
Pulmonary	889	67	337	37.9	1		1	
Extra-pulmonary	438	33	115	26.3	0.6 (0.5,0.8)	< 0.01	0.6 (0.5,0.8)	< 0.01
Total	1327	100	452	34.1				
Drug sensitivity								
Resistant to at least one drug	74	5.6	11	14.9	1		1	
Sensitive	1244	94.4	440	35.4	3.1 (1.6,6)	< 0.01	2.5 (1.3,5)	< 0.01
Total	1318	100	451	34.2				
Previous diagnosis								
No	1008	85.2	342	33.9	1			
Yes	175	14.8	70	40	1.3 (0.9,1.8)	0.12	–	–
Total	1183	100	412	34.8				
History of or current problem drug use**								
No	969	95.8	321	33.1	1			
Yes	42	4.2	33	78.6	7.4 (3.5,15.7)	< 0.01	–	–
Total	1011	100	354	35				
History of or current problem alcohol use**								
No	948	96.5	322	34	1			
Yes	34	3.5	23	67.7	4.1 (2,8.4)	< 0.01	–	–
Total	982	100	345	35.1				
History of or current homelessness**								
No	985	96.8	342	34.7	1			
Yes	33	3.2	16	48.5	1.8 (0.9,3.5)	0.11	–	–
Total	1018	100	358	35.2				
History of or currently in prison**								
No	932	95.9	316	33.9	1			
Yes	40	4.1	28	70	4.5 (2.3,9.1)	< 0.01	–	–
Total	972	100	344	35.4				

*Foreign-born only

**Missing for the cases notified in 2007 and 2008, and for half of those notified in 2009

Considering the foreign-born, the percentage clustered retrospectively increased with increasing time since entering the UK from 22 to 29% for those present for < 1 and at least 10 years respectively, although the difference was not statistically significant.

Extrapulmonary cases were less likely than pulmonary cases to be clustered retrospectively (26% vs 38%, OR: 0.6, 95% CI: 0.5–0.8). Drug-sensitive cases were more likely to be clustered retrospectively, compared to those resistant to at least one drug (35% vs 15%, OR: 3.1, 95% CI: 1.6–6.0). History of/current problem drug use, problem alcohol use and imprisonment were each associated with retrospective clustering (OR of 7.4 (95% CI: 3.5–15.7), 4.1 (95% CI: 2.0–8.4) and 4.5 (95% CI: 2.3–9.1) respectively, *p* < 0.01).

Multivariable analyses showed that retrospectively clustered cases were less likely to be female than male (aOR = 0.8, 95% CI: 0.6–1.0, *p* = 0.03), aged 45–64 or ≥ 65 years than 0–14 years (aOR = 0.4 (95% CI: 0.2–0.9) and 0.3 (95% CI: 0.1–0.7) respectively, and extrapulmonary than pulmonary (aOR = 0.6, 95% CI: 0.5–0.8). They were also less likely to be born in Europe, the East Mediterranean, Africa, South Asia and East/Southeast Asia, than UK-born (e.g. aOR = 0.4 (95%CI: 0.3–0.5) considering cases born in South Asia, compared to UK-born). Drug-sensitive cases were more likely than drug-resistant cases to be retrospectively clustered (aOR = 2.5, 95% CI:1.3–5.0).

### Analyses of “who’s clustered with whom”

**Fig. 3:**
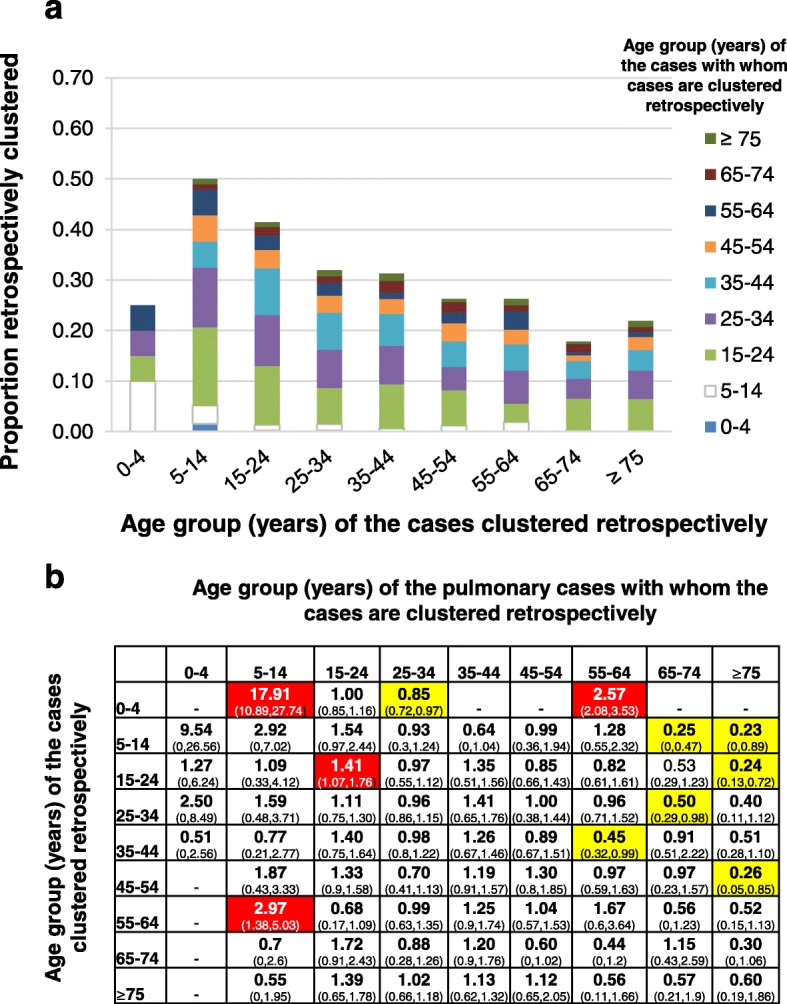
Analysis of the age-groups of the pulmonary cases with whom cases notified during 2009–11 were clustered retrospectively. **a** Proportions of cases in each age group who were retrospectively clustered with pulmonary cases in other age groups. **b** Retrospective clustering ratio for cases in each age group. Yellow and red cells show less and more retrospective clustering respectively with pulmonary cases in a given age group than might be expected, with 95% confidence intervals in parentheses. Dashes indicate ratios for which the ratio was zero and confidence intervals could not be calculated using the bootstrapping approach. Unshaded cells show ratios for which there is neither more nor less retrospective clustering than might be expected

At least 40% of retrospective clustering in each age group with pulmonary cases was with cases aged under 35 years (Fig. 3a). More retrospective clustering than expected occurred between 0-4 year olds and 5–14 year old pulmonary cases (RCR of 17.9 (95% CI: 10.9–27.8) and between 0-4 year olds and 55–64 year old pulmonary cases (RCR of 2.6 (95% CI: 2.1–3.5), Fig. 3b), between 15-24 year olds and pulmonary cases in the same age group (RCR of 1.4 (95% CI: 1.1–1.8) and between 55-64 year olds and 5–14 year old pulmonary cases (RCR of 3.0, 95% CI: 1.4–5.0). However, less retrospective clustering than expected occurred between several younger age groups and older cases (RCR of 0.23 (95% CI: 0–0.89) between 5-14 year olds and ≥ 75 year old pulmonary cases, and 0.24 (95% CI: 0.13–0.72) between 15-24 year olds and ≥ 75 year old pulmonary cases).

**Fig. 4:**
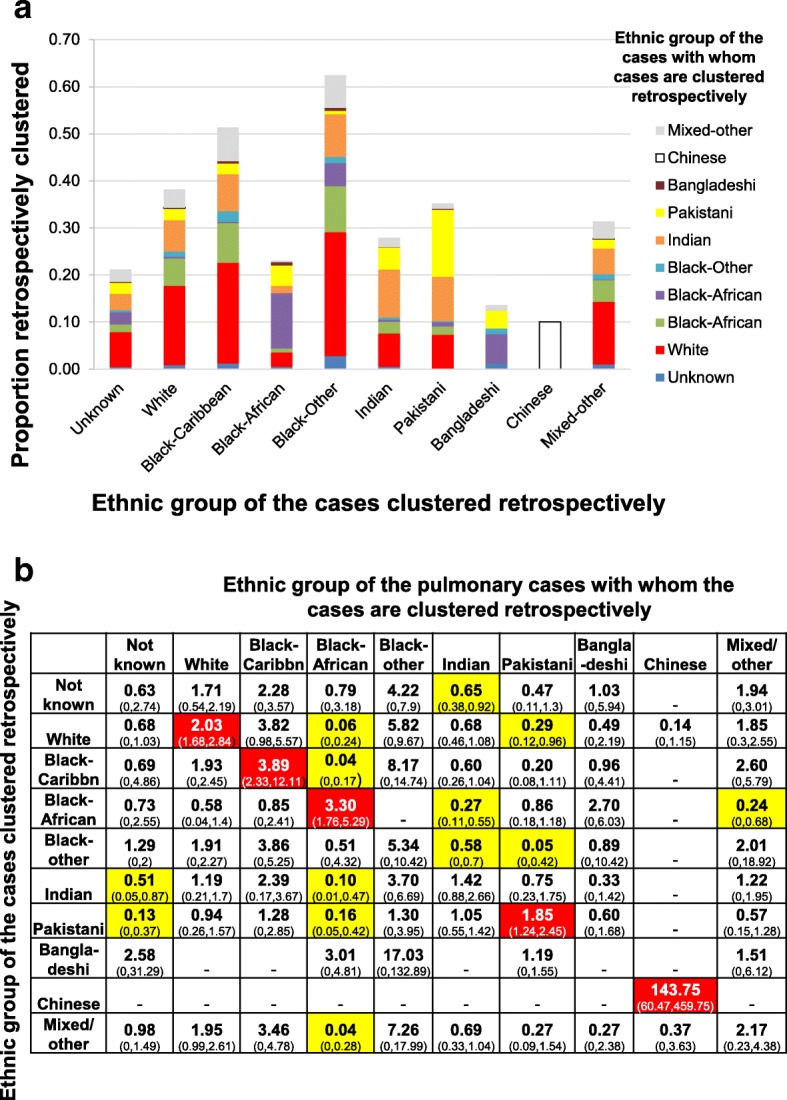
Analysis of the ethnic groups of the pulmonary cases with whom cases notified during 2009–11 were clustered retrospectively. **a** Proportions of cases in each ethnic group who were retrospectively clustered with pulmonary cases in other ethnic groups. **b** Retrospective clustering ratio for cases in each ethnic group, with 95% confidence intervals in parentheses. Dashes indicate ratios for which the ratio was zero and confidence intervals could not be calculated using the bootstrapping approach. See the caption to Fig. 3 for the interpretation of the colour coding

A large percentage (40–50%) of the retrospective clustering in several ethnic groups (the white, Black Caribbean, Black Other and “Mixed other”) occurred with cases in the white ethnic group (Fig. 4a). More retrospective clustering than expected occurred with pulmonary cases in the same ethnic group for the white, Black Caribbean, Black African, Pakistani and Chinese groups (RCR of 2.03 (95% CI: 1.7–2.8), 3.9 (95% CI: 2.3–12.1), 3.3 (95% CI: 1.8–5.3), 1.8 (95% CI: 1.2–2.4) and 143.7 (95% CI: 60.5–459.7) respectively). There was also less retrospective clustering than expected between several ethnic groups and the Black African, Indian and Pakistani groups.

**Fig. 5:**
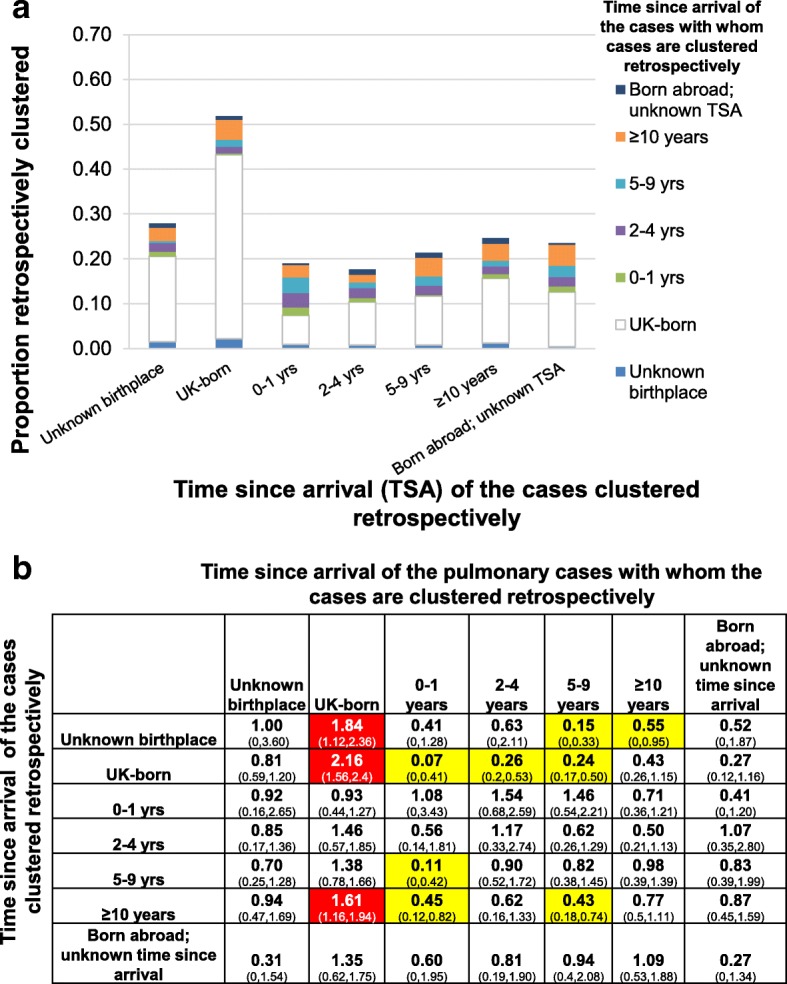
Analysis of the time since arrival of the pulmonary cases with whom cases notified during 2009–11 were clustered retrospectively. **a** Proportions of cases with different time since arrival (TSA) who were retrospectively clustered with pulmonary cases with other times since arrival. **b** Retrospective clustering ratio for cases with different time since arrivals, with 95% confidence intervals in parentheses. Dashes indicate ratios for which the ratio was zero and confidence intervals could not be calculated using the bootstrapping approach. See the caption to Fig. 3 for the interpretation of the colour coding

The greatest proportion of the retrospective clustering among immigrants, irrespective of their time since arrival in England or birthplace was with UK-born cases (Fig. 5a). There was more retrospective clustering than expected with UK-born pulmonary cases among those with an unknown birthplace, UK-born cases and those present in the UK for at least 10 years (RCR: 1.8 (95% CI: 1.1–2.4), 2.2 (95% CI: 1.6–2.4) and 1.6 (95% CI: 1.2–1.9) respectively). Conversely, less retrospective clustering than expected occurred between the UK-born and cases who had arrived within 10 years previously (RCR of 0.07 (95% CI: 0–0.41), 0.26 (95% CI: 0.2–0.53) and 0.24 (95% CI: 0.17–0.50) for those present in the UK for 0–1, 2–4 and 5–9 years respectively). There was also less retrospective clustering than expected between cases present in the UK for 5 or more years and recent immigrants with pulmonary TB (RCR of 0.11 (95% CI: 0–0.42) and 0.45 (95% CI: 0.12–0.82) for those present in the UK for 5–9 and at least 10 years respectively).

### Sensitivity analyses

For most age, ethnic groups and time since arrival in the UK, the percentage retrospectively clustered increased slightly as the time window used to identify a matching isolate lengthened, although the confidence intervals widened (Additional file 1: Figure S1). For 35–44 year olds for example, it increased from 31% (95% CI: 25–38) using a 2 year time window, to 37% (95% CI: 29–45) and 44% (95% CI: 33–55) using a three and a 4 year time window respectively. However, the age, ethnic group and time since arrival of the cases with whom cases were retrospectively clustered were similar when the time window used to calculate retrospective clustering increased (Additional file 1: Figure S2).

Increasing the time window to 3 years led to more retrospective clustering than expected occurring only between 5-14 year olds and 15–24 year old pulmonary cases (RCR: 2.4 (95% CI: 1.0–3.5)) and between 55-64 year olds and pulmonary cases in the same age group (RCR: 2.3 (95% CI: 1.3–4.4)), with more retrospective clustering than expected occurring only for the latter using a 4 year time window (Additional file 1: Figure S3). The age groups for which less clustering than expected occurred were similar for all time windows (Additional file 1: Figure S3).

Findings by ethnic group were similar using a two and 3 year window to define retrospective clustering; using a 4 year time window, more retrospective clustering than expected was seen only with pulmonary cases in the same ethnic group for the white and Pakistani groups, and with those in the Mixed/other group with cases in the Black-other group (Additional file 1: Figure S4).

Considering the cases by time since arrival in the UK, the groups for which the RCR was higher or lower than expected were similar for all time windows used for defining retrospective clustering (Additional file 1: Figure S5). For a 4 year time window, more retrospective clustering than expected also occurred among the UK-born or unknown birthplace and pulmonary cases with an unknown time since arrival (RCR: 1.82 (95% CI: 1.53–2.19) and 5.44 (95% CI: 4.40–7.22)).

## Discussion

Our analyses appear to be the first to quantify the amount of clustering between different population groups in a high TB incidence area in England using molecular data. We found that retrospective clustering with pulmonary cases between some ethnic groups was over two-fold greater than expected, and more clustering than expected occurred between 15-24 year olds and between UK-born or long-term immigrants with the UK-born. The findings provide insight into transmission patterns between different groups and possible ways of prioritising contact tracing in high incidence areas.

The definition of clustering used here, i.e. the proportion of cases who were clustered with pulmonary cases up to 2 years previously, differs from that used in other molecular epidemiological studies in the UK and has two advantages. By definition, cases cannot be retrospectively clustered with cases notified after them, who could have been their secondary cases. Consequently, the proportion retrospectively clustered is more closely related to the proportion of disease that is attributable to recent transmission than is the overall proportion clustered. Second, it eliminates some bias that occurs for other clustering definitions, such as the “n-1” method, for which cases notified at different times have different follow-up periods for assessing clustering. Using the retrospective method, the same time period for each isolate is used to identify its match, and, as suggested by our analyses (Table 3), the proportion retrospectively clustered within a given period will probably be relatively insensitive to the time period spanned by the dataset, if there are no changes in the amount of ongoing transmission.

**Table 3:** Estimates of the amount of disease attributable to recent transmission calculated using the “n-1” method and retrospective clustering with cases up to two years beforehand, using all cases notified within different time periods during 2007–11

Time period	Number of cases clustered, excluding the first case	Number of cases notified during the study period	Number retrospectively clustered with cases up to 2 years previously	Number of cases with onset more than two years after the start of the study period	% due to recent transmission based on:	
					“n-1” formula	Retrospective clustering
2007–11	734	2159	452	1329	34% (32,36)	34% (31,37)
2007–10	554	1721	302	891	32% (30,34)	34% (31,37)
2007–9	393	1291	156	461	30% (28,33)	34% (30,38)

Numbers in parentheses denote (exact binomial) 95% confidence intervals

Only the cases who had onset two or more years after the start of the study period were used in the denominator for the retrospective clustering percentage

The size of the bias resulting from differing follow-up periods for the “n-1” method, and differences between the method’s estimates and the proportion retrospectively clustered depends on the study period duration (Table 3). This results from the fact that the denominator used in the percentage clustered for the “n-1” method includes all cases notified in the study period, and cases notified early in the period but infected 2 years previously would be mistakenly attributed to reactivation. The proportion of cases affected by the misclassification decreases as the study period lengthens, as the proportion of cases for whom it becomes possible to identify a case with a matching genotype increases.

Our finding that the proportion retrospectively clustered increases with the time window used to assess clustering is consistent with that from other studies [15], resulting from the increased probability of both the source and secondary case being notified during the study period. However, the confidence intervals on both the proportion retrospectively clustered and the retrospective clustering ratio widened with the increased time window, reducing the ability to detect retrospective clustering that is higher than expected. These widening confidence intervals follow from the data-loss that occurs with the retrospective clustering approach, which increases with longer retrospective time periods considered. For example, as defined here, when calculating the proportion clustered retrospectively, the first 2 years of notified cases were excluded from the denominator, increasing to exclusion of 4 years of notified cases when using a 4 year window to define retrospective clustering.

We used the retrospective clustering ratio to estimate whether the clustering seen between two population groups was more or less than that expected, based on the group’s size among notified cases. Analogous statistics have been used in social contact surveys to compare the amount of contact between different populations. Such statistics may be biased and overestimate clustering between population groups if strain-typing of isolates was done preferentially for certain cases, such as those involved in contact investigations. The time required to obtain results from strain-typing data means that strain-typing is unlikely to have been carried out preferentially for cases involved in contact investigations [16].

However, we probably underestimated the proportion of cases in some population groups that were clustered, as genotyping was only conducted for culture-positive cases, who comprised 57% of cases during the study period and sampling a proportion of the data leads to underestimates in the amount of clustering [17, 18]. One study from The Netherlands [19] found that a significantly larger proportion of cases without a typed isolate had a confirmed recent epidemiological link and could be presumed to have been recently infected than cases whose isolates had been typed (25% vs 18%, *P* < 0.01).

Undersampling of some population groups, such as the UK-born (Table 1) for whom genotyping data were available for fewer than half of the cases, could have also affected the retrospective clustering ratios, which considers the pulmonary cases with whom cases are clustered retrospectively and population groups that they come from. If those undersampled cases had pulmonary tuberculosis and they transmitted to other population groups, the retrospective clustering ratio for the latter groups with the undersampled groups could be underestimated. The size of the underestimate may be relatively small, since over half of those without genotype data had extrapulmonary tuberculosis.

Contact tracing seeks to seeks to identify and diagnose contacts of infectious cases and is highlighted as a key component for tuberculosis control by the national tuberculosis strategy. The largest impact on case finding will be obtained by focusing on the groups that are likely to have the highest yield from case-finding. Estimates of the retrospective clustering ratio can contribute to this by indicating which population groups may give the highest yield for cases in a given population group. For some ethnic groups, there was more retrospective clustering than expected with pulmonary cases in their own ethnic group, suggesting that the source of infection, and potentially, the greatest case-finding yield, may be obtained from contacts in the same ethnic group. Analogous conclusions apply to our finding of more retrospective clustering than expected between 15-24 year olds and pulmonary cases in the same age group and between UK-born cases and immigrants who had arrived at least 10 years previously and pulmonary UK-born cases. Prioritising contact tracing for cases in particular groups on those most likely to cluster retrospectively with them could speed up case-finding and, by shortening the time during which cases are infectious, improve TB control.

More retrospective clustering than expected occurred between 55-64 year old cases and 5–14 year old pulmonary cases. Since many 55–64 year old cases were probably infected many years previously, this finding may follow from several study limitations. For example, cases may be retrospectively clustered with cases who are not their source of infection, since clustering may occur if a common genotype has been circulating in the population. Also, since retrospective clustering was defined using the notification date as a proxy for the onset date, the outcome could have occurred if, as is plausible, the time from onset to diagnosis was shorter for 0–4 than 55 year old cases. Another limitation is that if a case’s infectious source lived outside the study region or had been notified over 2 years before the case of interest, they would not contribute to calculations of the retrospective clustering proportion.

A smaller proportion of extrapulmonary than pulmonary cases were retrospectively clustered with pulmonary cases, even after adjusting for the birthplace and other factors. Other studies, which considered the overall proportion of cases that were clustered and, unlike our estimates, had the biases described above, had similar findings. Our finding may be attributable to several factors, including undersampling of extrapulmonary cases, due to the facts that the genotype of culture-negative cases was not determined and most culture-negative cases are extrapulmonary. Also, due to the non-specificity of symptoms, extrapulmonary cases are more difficult to diagnose than are pulmonary cases. This may lead to increased diagnostic delays among extrapulmonary cases and reduce the chance of finding their source of infection or cases who shared the same genotype within the 2 year period for retrospective clustering.

It is reassuring that our estimates of the proportion of disease attributable to recent transmission are comparable to those found elsewhere in Western Europe. Also, our findings of the amount of clustering among immigrants is consistent with those elsewhere in England. The finding that there was neither more nor less retrospective clustering than expected between recent immigrants and other immigrant groups is consistent with hypotheses that disease among recent immigrants is attributable to infection acquired abroad. The finding of more retrospective clustering than expected for those who had arrived at least 10 years previously and pulmonary UK-born cases suggests that with increasing time spent in the UK, acquiring infection from UK-born cases becomes increasingly likely.

## Conclusions

In conclusion, our study provides important insight into both the amount of *M tuberculosis* transmission in one high incidence area in England and the amount of transmission between different age, ethnic and immigrant groups. These findings are relevant for the recent collaborative tuberculosis strategy which highlighted contact tracing and reducing diagnostic delay as important for reducing tuberculosis incidence in England. Prioritising contact tracing for cases in particular groups on those most likely to cluster retrospectively with them could speed up case finding and, by shortening the time during which cases are infectious, improve TB control.

Further studies are needed to determine whether our findings are generalizable nationally and to high incidence areas elsewhere in England. The future accumulation of long-term data from whole-genome sequencing, which was introduced routinely in England in 2017 [20] and has a higher resolution than does 24-locus MIRU-VNTR, should provide further insight into *M tuberculosis* transmission patterns in England.

## Additional file

Additional file 1: This contains the results of the sensitivity of the retrospective clustering analyses to the size of the time window used. (PDF 291 kb)

## Abbreviations

ETS: Enhanced tuberculosis surveillance
LRT: Likelihood ratio test
MIRU-VNTR: Mycobacterial Interspersed Repetitive Unit-Variable Number Tandem Repeats
OR: Odds ratio
RCR: Retrospective clustering ratio
TB: Tuberculosis
