# Supplementary material for: Mycobacterium tuberculosis transmission in an ethnically-diverse high incidence region in England, 2007–11
Source: BMC Infect Dis. 2019 Jan 7;19:26. doi: 10.1186/s12879-018-3585-8 (PMC6323781; doi:10.1186/s12879-018-3585-8)
Supplement: Supplementary file 1 — This contains the results of the sensitivity of the retrospective clustering analyses to the size of the time window used. (PDF 291 kb) [file 12879_2018_3585_MOESM1_ESM.pdf]

Mycobacterium tuberculosis transmission in an ethnically-diverse high incidence region in England, 2007-11

Supplementary Material

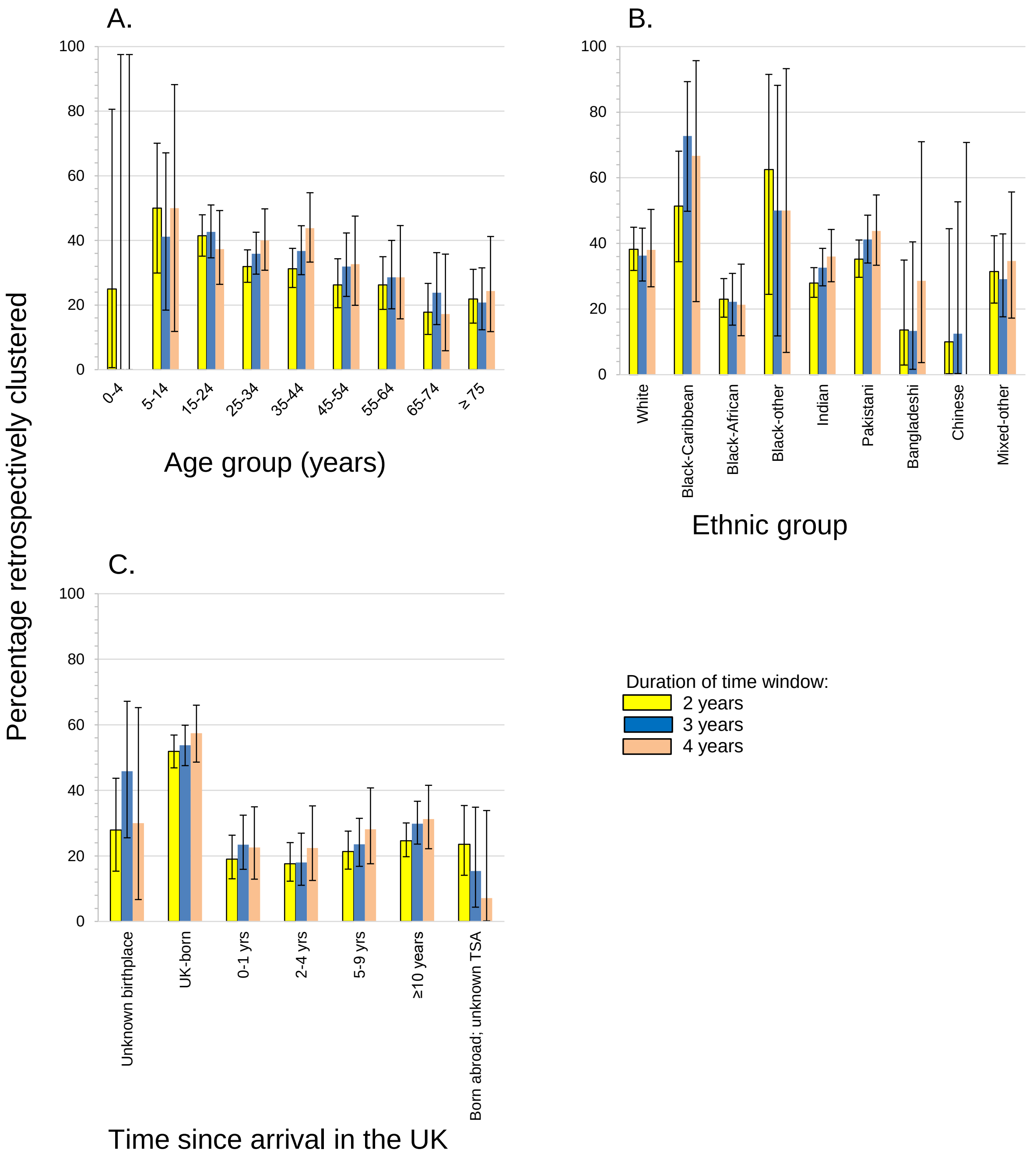

Figure S1: Percentage of cases by A. age, B. ethnic and C. immigrant group notified during 2009-11 that were retrospectively clustered with pulmonary cases, calculated using 2, 3 and 4 year time windows.

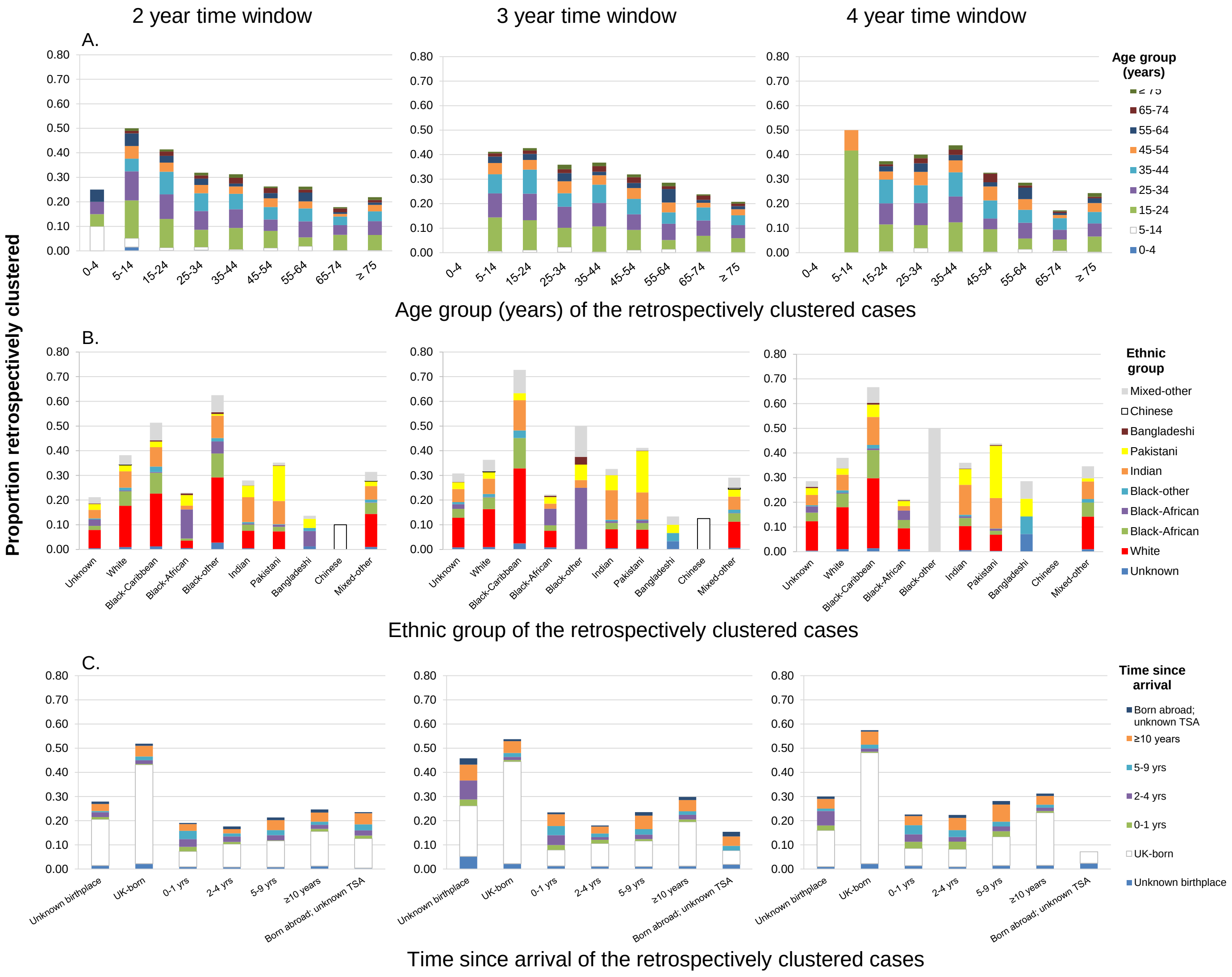

Figure S2: Proportions of cases notified during 2009-2011 in each A. age group B. ethnic group and C. cases with different time since arrival who were retrospectively clustered with pulmonary cases in other age groups, ethnic groups and cases with different time since arrival, calculated using time windows of 2, 3 and 4 years.

Age group (years) of the pulmonary cases with whom the cases are clustered retrospectively

Age group (years) of the cases clustered retrospectively

2 year time window

	0-4	5-14	15-24	25-34	35-44	45-54	55-64	65-74	≥75
0-4	-	17.91 (10.89,27.74)	1.00 (0.85,1.16)	0.85 (0.72,0.97)	-	-	2.57 (2.08,3.53)	-	-
5-14	9.54 (0,26.56)	2.92 (0,7.02)	1.54 (0.97,2.44)	0.93 (0.30,1.24)	0.64 (0,1.04)	0.99 (0.36,1.94)	1.28 (0.55,2.32)	0.25 (0,0.47)	0.23 (0,0.89)
15-24	1.27 (0,6.24)	1.09 (0.33,4.12)	1.41 (1.07,1.76)	0.97 (0.55,1.12)	1.35 (0.51,1.56)	0.85 (0.66,1.43)	0.82 (0.61,1.61)	0.53 (0.29,1.23)	0.24 (0.13,0.72)
25-34	2.50 (0,8.49)	1.59 (0.48,3.71)	1.11 (0.75,1.30)	0.96 (0.86,1.15)	1.41 (0.65,1.76)	1.00 (0.38,1.44)	0.96 (0.71,1.52)	0.50 (0.29,0.98)	0.40 (0.11,1.12)
35-44	0.51 (0,2.56)	0.77 (0.21,2.77)	1.40 (0.75,1.64)	0.98 (0.8,1.22)	1.26 (0.67,1.46)	0.89 (0.67,1.51)	0.45 (0.32,0.99)	0.91 (0.51,2.22)	0.51 (0.28,1.10)
45-54	-	1.87 (0.43,3.33)	1.33 (0.90,1.58)	0.70 (0.41,1.13)	1.19 (0.91,1.57)	1.30 (0.8,1.85)	0.97 (0.59,1.63)	0.97 (0.23,1.57)	0.26 (0.05,0.85)
55-64	-	2.97 (1.38,5.03)	0.68 (0.17,1.09)	0.99 (0.63,1.35)	1.25 (0.9,1.74)	1.04 (0.57,1.53)	1.67 (0.6,3.64)	0.56 (0,1.23)	0.52 (0.15,1.13)
65-74	-	0.70 (0,2.60)	1.72 (0.91,2.43)	0.88 (0.28,1.26)	1.20 (0.9,1.76)	0.60 (0,1.02)	0.44 (0,1.20)	1.15 (0.43,2.59)	0.30 (0,1.06)
≥75	-	0.55 (0,1.95)	1.39 (0.65,1.78)	1.02 (0.66,1.18)	1.13 (0.62,1.32)	1.12 (0.65,2.05)	0.56 (0.11,1.66)	0.57 (0.21,1.90)	0.60 (0.19,1.86)

3 year time window

	0-4	5-14	15-24	25-34	35-44	45-54	55-64	65-74	≥75
0-4	-	-	-	-	-	-	-	-	-
5-14	-	0.64 (0,10.14)	1.67 (0.44,4.61)	0.95 (0,1.11)	1.15 (0,1.36)	1.06 (0,2.72)	0.78 (0,2.71)	0.39 (0,0.58)	0.18 (0,1.23)
15-24	0.75 (0,5.82)	0.93 (0.21,4.87)	1.42 (0.96,1.84)	1.02 (0.6,1.13)	1.40 (0.64,1.60)	0.88 (0.57,1.64)	0.7 (0.53,1.69)	0.41 (0.10,0.90)	0.26 (0.14,0.82)
25-34	1.46 (0,4.21)	2.39 (1.04,3.50)	1.09 (0.72,1.39)	0.97 (0.82,1.30)	0.95 (0.73,1.18)	1.27 (0.43,1.65)	1.11 (0.7,1.57)	0.54 (0.23,0.92)	0.57 (0.14,1.12)
35-44	-	0.46 (0.07,2.05)	1.4 (0.84,1.60)	1.05 (0.71,1.2)	1.26 (0.66,1.45)	0.98 (0.74,1.82)	0.45 (0.27,1.02)	0.77 (0.41,2.22)	0.43 (0.24,1.00)
45-54	-	1.38 (0.42,2.95)	1.29 (0.9,1.58)	0.79 (0.56,1.04)	1.21 (0.86,1.53)	1.31 (0.88,1.92)	0.77 (0.34,1.32)	0.92 (0.19,1.75)	0.39 (0.09,0.84)
55-64	-	2.07 (0,3.63)	0.65 (0.23,1.12)	0.93 (0.47,1.26)	0.99 (0.5,1.68)	1.33 (0.61,1.97)	2.33 (1.30,4.40)	0.50 (0,1.70)	0.57 (0,1.07)
65-74	1.87 (0,12.75)	0.60 (0,3.74)	1.32 (0.39,1.61)	1.04 (0.58,1.34)	1.37 (0.48,1.63)	0.76 (0.25,1.57)	0.62 (0,1.22)	0.91 (0.37,3.76)	0.25 (0,1.16)
≥75	-	0.42 (0,1.99)	1.37 (0.72,1.70)	1.02 (0.39,1.17)	1.19 (0,1.37)	1.10 (0.44,2.17)	0.74 (0.36,1.87)	0.63 (0.31,3.25)	0.40 (0.08,1.83)

4 year time window

	0-4	5-14	15-24	25-34	35-44	45-54	55-64	65-74	≥75
0-4	-	-	-	-	-	-	-	-	-
5-14	-	-	4.16 (0,5.55)	-	-	1.54 (0,9.72)	-	-	-
15-24	-	0.70 (0.09,5.35)	1.46 (0.89,2.32)	0.95 (0.43,1.10)	1.54 (0.78,1.75)	0.83 (0.38,1.50)	0.66 (0.39,1.88)	0.28 (0,0.39)	0.37 (0.2,10)
25-34	0.54 (0,2.51)	2.07 (0.72,3.53)	1.16 (0.59,1.43)	0.93 (0.78,1.28)	1.09 (0.84,1.36)	1.26 (0.63,1.62)	1.03 (0.64,1.71)	0.66 (0.18,1.06)	0.4 (0.08,0.95)
35-44	-	0.56 (0.06,2.35)	1.35 (0.85,1.55)	0.99 (0.72,1.16)	1.34 (0.84,1.55)	1.03 (0.71,1.82)	0.60 (0.38,1.21)	0.62 (0.33,1.75)	0.44 (0.16,1.04)
45-54	-	0.59 (0,3.15)	1.39 (0.75,1.93)	0.55 (0.24,0.92)	1.34 (0.91,1.81)	1.60 (0.76,3.20)	0.64 (0.12,1.78)	1.37 (0,2.12)	0.15 (0,0.47)
55-64	-	2.29 (0,3.96)	0.76 (0.11,1.27)	0.93 (0.55,1.22)	1.09 (0.77,1.71)	1.41 (0.7,2.25)	1.97 (1.12,4.2)	0.39 (0,0.93)	0.44 (0,0.84)
65-74	3.22 (0,33.41)	1.63 (0,11.37)	1.31 (0,1.51)	0.94 (0,1.53)	1.65 (0.52,4.15)	0.67 (0,0.89)	0.58 (0,1.05)	0.46 (0,0.63)	0.26 (0,5.30)
≥75	-	0.78 (0,2.21)	1.27 (0.76,2.55)	0.91 (0,1.08)	1.14 (0,1.43)	1.37 (0,2.46)	0.94 (0.36,2.76)	0.33 (0,1.81)	0.67 (0,3.02)

Figure S3: Retrospective clustering ratio for cases in each age group calculated using 2, 3 and 4 year time windows. Yellow and red cells show less and more retrospective clustering respectively with pulmonary cases in a given age group than might be expected, with 95% confidence intervals in parentheses. Dashes indicate ratios for which the ratio could not be calculated using the bootstrapping approach. Pink and pale yellow shading reflects ratios for which the RCR was bigger and smaller than expected respectively, but for which the estimate was inconclusive as the 95% confidence intervals overlapped 1.00.

Ethnic group of the cases clustered retrospectively

2 year time window

	Not known	White	Black-Caribbn	Black-African	Black-other	Indian	Pakistani	Bangla-deshi	Chinese	Mixed/ other
Not known	**0.63** (0,2.74)	**1.71** (0.54,2.19)	**2.28** (0,3.57)	**0.79** (0,3.18)	**4.22** (0,7.9)	**0.65** (0.38,0.92)	**0.47** (0.11,1.3)	**1.03** (0,5.94)	-	**1.94** (0,3.01)
White	**0.68** (0,1.03)	**2.03** (1.68,2.84)	**3.82** (0.98,5.57)	**0.06** (0,0.24)	**5.82** (0,9.67)	**0.68** (0.46,1.08)	**0.29** (0.12,0.96)	**0.49** (0,2.19)	**0.14** (0,1.15)	**1.85** (0.3,2.55)
Black-Caribbn	**0.69** (0,4.86)	**1.93** (0,2.45)	**3.89** (2.33,12.11)	**0.04** (0,0.17)	**8.17** (0,14.74)	**0.60** (0.26,1.04)	**0.20** (0.08,1.11)	**0.96** (0,4.41)	-	**2.60** (0,5.79)
Black-African	**0.73** (0,2.55)	**0.58** (0.04,1.40)	**0.85** (0,2.41)	**3.30** (1.76,5.29)	-	**0.27** (0.11,0.55)	**0.86** (0.18,1.18)	**2.70** (0,6.03)	-	**0.24** (0,0.68)
Black-other	**1.29** (0,2.00)	**1.91** (0,2.27)	**3.86** (0,5.25)	**0.51** (0,4.32)	**5.34** (0,10.42)	**0.58** (0,0.70)	**0.05** (0,0.42)	**0.89** (0,10.42)	-	**2.01** (0,18.92)
Indian	**0.51** (0.05,0.87)	**1.19** (0.21,1.7)	**2.39** (0.17,3.67)	**0.10** (0.01,0.47)	**3.70** (0,6.69)	**1.42** (0.88,2.66)	**0.75** (0.23,1.75)	**0.33** (0,1.42)	-	**1.22** (0,1.95)
Pakistani	**0.13** (0,0.37)	**0.94** (0.26,1.57)	**1.28** (0,2.85)	**0.16** (0.05,0.42)	**1.30** (0,3.95)	**1.05** (0.55,1.42)	**1.85** (1.24,2.45)	**0.60** (0,1.68)	-	**0.57** (0.15,1.28)
Bangla-deshi	**2.58** (0,31.29)	-	-	**3.01** (0,4.81)	**17.03** (0,132.89)	-	**1.19** (0,1.55)	-	-	**1.51** (0,6.12)
Chinese	-	-	-	-	-	-	-	-	**143.75** (60.47,459.75)	-
Mixed/ other	**0.98** (0,1.49)	**1.95** (0.99,2.61)	**3.46** (0,4.78)	**0.04** (0,0.28)	**7.26** (0,17.99)	**0.69** (0.33,1.04)	**0.27** (0.09,1.54)	**0.27** (0,2.38)	**0.37** (0,3.63)	**2.17** (0.23,4.38)

3 year time window

	Not known	White	Black-Caribbn	Black-African	Black-other	Indian	Pakistani	Bangla-deshi	Chinese	Mixed/ other
Not known	**0.84** (0,2.18)	**1.78** (0.86,2.28)	**2.81** (0,4.19)	**0.40** (0,2.25)	**5.35** (0,9.28)	**0.68** (0.42,0.99)	**0.41** (0.13,1.19)	**0.57** (0,4.00)	-	**1.98** (0,2.83)
White	**0.76** (0,1.07)	**1.98** (1.65,2.89)	**3.40** (1.07,4.86)	**0.04** (0,0.29)	**6.59** (0,10.82)	**0.67** (0.37,0.89)	**0.34** (0.14,1.12)	-	**0.53** (0,4.51)	**2.30** (0.18,2.99)
Black-Caribbn	**0.99** (0,1.39)	**1.90** (0.23,2.23)	**3.88** (2.24,8.41)	-	**8.13** (0,13.3)	**0.67** (0.44,1.68)	**0.18** (0.08,1.60)	-	-	**2.42** (0,3.99)
Black-African	**1.07** (0.22,4.74)	**1.40** (0.13,2.00)	**2.46** (0,4.19)	**1.97** (0.77,5.07)	-	**0.36** (0.09,0.49)	**0.55** (0,0.73)	**1.98** (0,5.34)	-	**0.41** (0,0.72)
Black-other	-	-	-	**3.29** (0,4.67)	-	**0.25** (0,0.36)	**0.55** (0,0.85)	**4.79** (0,8.72)	-	**4.55** (0,22.72)
Indian	**0.43** (0.04,0.77)	**1.10** (0.26,1.61)	**2.08** (0.24,3.30)	**0.13** (0.01,0.55)	**3.86** (0,7.08)	**1.44** (0.91,2.62)	**0.83** (0.24,1.80)	**0.23** (0,1.05)	-	**1.34** (0,2.21)
Pakistani	**0.26** (0,0.67)	**0.86** (0.25,1.43)	**1.61** (0.36,3.00)	**0.17** (0.03,0.49)	**1.68** (0,4.38)	**1.04** (0.54,1.39)	**1.86** (1.25,2.44)	**0.39** (0,1.18)	-	**0.48** (0.08,1.08)
Bangla-deshi	**6.97** (0,34.8)	-	-	-	**47.06** (0,215.81)	-	**1.14** (0,1.73)	-	-	**4.47** (0,7.73)
Chinese	-	-	-	-	-	-	-	-	**166.2** (65.84,348.98)	-
Mixed/ other	**0.67** (0,1.02)	**1.65** (0.11,2.25)	**2.77** (0,4.01)	**0.04** (0,0.34)	**8.62** (0,27.44)	**0.73** (0.33,1.23)	**0.48** (0.11,2.11)	-	**1.96** (0,15.57)	**2.65** (0,6.67)

4 year time window

	Not known	White	Black-Caribbn	Black-African	Black-other	Indian	Pakistani	Bangla-deshi	Chinese	Mixed/ other
Not known	**0.46** (0,0.94)	**1.86** (0.29,2.33)	**2.89** (0,5.06)	**0.60** (0,3.02)	**2.90** (0,6.18)	**0.58** (0.38,1.15)	**0.45** (0.07,1.24)	**1.30** (0,4.70)	-	**1.46** (0,2.44)
White	**0.78** (0,1.13)	**2.03** (1.69,3.05)	**3.67** (0.71,5.23)	**0.05** (0,0.34)	**5.07** (0,8.93)	**0.65** (0.35,0.93)	**0.30** (0.11,1.17)	**0.16** (0,1.03)	-	**2.04** (0,2.67)
Black-Caribbn	**0.60** (0,1.12)	**1.92** (0,4.42)	**4.02** (0,6.22)	**0.07** (0,0.21)	**3.86** (0,8.65)	**0.67** (0,2.19)	**0.34** (0,1.64)	**0.85** (0,2.90)	-	**1.75** (0,2.78)
Black-African	**1.32** (0.35,7.77)	**1.81** (0.19,2.23)	**3.92** (0,6.05)	**1.22** (0.46,5.47)	-	**0.33** (0,0.46)	**0.44** (0,0.59)	**0.96** (0,1.71)	-	**0.43** (0,0.64)
Black-other	-	-	-	-	-	-	-	-	-	**18.85** (14.54,25.62)
Indian	**0.52** (0.05,0.92)	**1.23** (0.26,1.76)	**2.23** (0.09,3.51)	**0.13** (0.01,0.62)	**3.54** (0,6.91)	**1.32** (0.84,2.52)	**0.81** (0.17,1.89)	**0.37** (0,1.76)	-	**1.19** (0,1.95)
Pakistani	**0.20** (0,0.73)	**0.68** (0.18,1.46)	**0.89** (0,2.80)	**0.12** (0.02,0.41)	**0.65** (0,3.94)	**1.11** (0.39,1.45)	**2.21** (1.59,2.91)	**0.57** (0,2.03)	-	**0.26** (0.04,0.85)
Bangla-deshi	**6.83** (0,33.53)	-	-	-	**47.46** (0,134.7)	-	**1.14** (0,1.70)	-	-	**4.38** (0,7.23)
Chinese	-	-	-	-	-	-	-	-	-	-
Mixed/ other	**0.79** (0,1.10)	**1.71** (0,2.02)	**3.76** (0,5.03)	-	**7.63** (0,49.87)	**0.82** (0.61,2.49)	**0.16** (0,1.41)	-	-	**2.61** (0,10.30)

Figure S4: Retrospective clustering ratio for cases in each ethnic group, calculated using 2, 3 and 4 year time windows. Yellow and red cells show less and more retrospective clustering respectively with pulmonary cases in a given ethnic group than might be expected, with 95% confidence intervals in parentheses. Dashes indicate ratios for which the ratio could not be calculated using the bootstrapping approach. Pink and pale yellow shading reflects ratios for which the RCR was bigger and smaller than expected respectively, but for which the estimate was inconclusive as the 95% confidence intervals overlapped 1.00.

Time since arrival of the pulmonary cases with whom the cases are clustered retrospectively

Time since arrival of the cases clustered retrospectively

2 year time window

	Unknown birthplace	UK-born	0-1 years	2-4 years	5-9 years	≥10 years	Born abroad; unknown time since arrival
Unknown birthplace	**1.00** (0,3.60)	**1.84** (1.12,2.36)	**0.41** (0,1.28)	**0.63** (0,2.11)	**0.15** (0,0.33)	**0.55** (0,0.95)	**0.52** (0,1.87)
UK-born	**0.81** (0.59,1.20)	**2.16** (1.56,2.40)	**0.07** (0,0.41)	**0.26** (0.2,0.53)	**0.24** (0.17,0.50)	**0.43** (0.26,1.15)	**0.27** (0.12,1.16)
0-1 yrs	**0.92** (0.16,2.65)	**0.93** (0.44,1.27)	**1.08** (0,3.43)	**1.54** (0.68,2.59)	**1.46** (0.54,2.21)	**0.71** (0.36,1.21)	**0.41** (0,1.20)
2-4 yrs	**0.85** (0.17,1.36)	**1.46** (0.57,1.85)	**0.56** (0.14,1.81)	**1.17** (0.33,2.74)	**0.62** (0.26,1.29)	**0.50** (0.21,1.13)	**1.07** (0.35,2.80)
5-9 yrs	**0.70** (0.25,1.28)	**1.38** (0.78,1.66)	**0.11** (0,0.42)	**0.90** (0.52,1.72)	**0.82** (0.38,1.45)	**0.98** (0.39,1.39)	**0.83** (0.39,1.99)
≥10 years	**0.94** (0.47,1.69)	**1.61** (1.16,1.94)	**0.45** (0.12,0.82)	**0.62** (0.16,1.33)	**0.43** (0.18,0.74)	**0.77** (0.5,1.11)	**0.87** (0.45,1.59)
Born abroad; unknown time since arrival	**0.31** (0,1.54)	**1.35** (0.62,1.75)	**0.60** (0,1.95)	**0.81** (0.19,1.90)	**0.94** (0.40,2.08)	**1.09** (0.53,1.88)	**0.27** (0,1.34)

3 year time window

	Unknown birthplace	UK-born	0-1 years	2-4 years	5-9 years	≥10 years	Born abroad; unknown time since arrival
Unknown birthplace	**2.02** (0,7.56)	**1.23** (0.62,1.93)	**0.63** (0,1.64)	**1.57** (0,3.34)	-	**0.74** (0,1.28)	**0.91** (0,2.78)
UK-born	**0.79** (0.59,1.28)	**2.16** (1.50,2.40)	**0.14** (0,0.77)	**0.22** (0.12,0.41)	**0.24** (0.18,0.49)	**0.46** (0.28,1.21)	**0.26** (0.09,1.07)
0-1 yrs	**1.02** (0.35,2.59)	**0.77** (0.32,1.05)	**0.96** (0,3.46)	**1.57** (0.79,2.84)	**1.33** (0.59,1.92)	**1.04** (0.32,1.55)	**0.55** (0,1.89)
2-4 yrs	**1.17** (0.54,1.97)	**1.45** (0.59,1.94)	**0.93** (0.30,2.70)	**0.56** (0.29,1.53)	**0.68** (0.18,1.31)	**0.80** (0.29,1.38)	**0.39** (0,1.23)
5-9 yrs	**0.79** (0,1.15)	**1.22** (0.34,1.71)	**0.38** (0.07,0.92)	**0.71** (0.44,1.89)	**0.81** (0.43,1.96)	**1.21** (0.66,1.78)	**1.02** (0.47,2.55)
≥10 years	**0.79** (0.49,1.41)	**1.68** (1.06,1.99)	**0.36** (0.13,0.77)	**0.58** (0.18,1.31)	**0.41** (0.19,0.75)	**0.79** (0.43,1.38)	**0.74** (0.32,1.63)
Born abroad; unknown time since arrival	**2.38** (0,6.48)	**1.01** (0,1.77)	-	-	**1.03** (0,2.68)	**1.29** (0,4.73)	**2.03** (0,14.51)

4 year time window

	Unknown birthplace	UK-born	0-1 years	2-4 years	5-9 years	≥10 years	Born abroad; unknown time since arrival
Unknown birthplace	**0.53** (0,5.30)	**1.37** (0.60,2.29)	**0.75** (0,4.03)	**1.83** (0,4.42)	**0.28** (0,0.70)	**0.68** (0,1.13)	**0.55** (0,1.30)
UK-born	**0.72** (0.5,0.97)	**2.19** (1.60,2.43)	**0.07** (0,0.43)	**0.21** (0.15,0.49)	**0.23** (0.12,0.52)	**0.47** (0.29,1.23)	**0.19** (0.07,0.94)
0-1 yrs	**1.14** (0,4.45)	**0.87** (0,1.15)	**1.29** (0,4.38)	**1.25** (0.76,4.05)	**1.36** (0.35,2.84)	**0.84** (0,1.07)	**0.54** (0,0.80)
2-4 yrs	**0.81** (0,1.55)	**0.89** (0.13,1.17)	**1.49** (0.69,3.75)	**0.78** (0.35,2.06)	**1.03** (0.38,1.79)	**1.13** (0.48,1.62)	**1.01** (0,2.82)
5-9 yrs	**0.88** (0.36,2.14)	**1.16** (0.11,1.77)	**0.91** (0.12,1.91)	**0.63** (0.38,2.53)	**0.58** (0,0.83)	**1.28** (0.73,2.25)	**0.88** (0.21,2.77)
≥10 years	**0.90** (0.59,1.43)	**1.91** (1.33,2.18)	**0.25** (0.06,0.70)	**0.39** (0.18,0.94)	**0.33** (0.11,0.79)	**0.58** (0.33,1.13)	**0.57** (0.26,1.44)
Born abroad; unknown time since arrival	**5.44** (4.40,7.22)	**1.82** (1.53,2.19)	-	-	-	-	-

Figure S5: Retrospective clustering ratio for cases with different times of arrival, calculated using 2, 3 and 4 year time windows. Yellow and red cells show less and more retrospective clustering respectively with pulmonary cases with a given time since arrival than might be expected, with 95% confidence intervals in parentheses. Dashes indicate ratios for which the ratio could not be calculated using the bootstrapping approach. Pink and pale yellow shading reflects ratios for which the RCR was bigger and smaller than expected respectively, but for which the estimate was inconclusive as the 95% confidence intervals overlapped 1.00.
